# Amorphous Cu-FeOOH Clusters on Oxygen-Deficient Fe-Doped BiOCl Nanoflowers for Efficient Photo-Fenton Degradation of Levofloxacin

**DOI:** 10.3390/nano16191207

**Published:** 2026-09-24

**Authors:** Hailin Zhang, Jin Cao, Xin Liu, Di Wei, Shuangpeng Yan, Xiangmei Wang, Hao Liu, Han Shi, Xinkun Ren, Shanyuan Niu

**Affiliations:** Jiangsu Key Laboratory of Functional Metamaterials, College of Engineering and Applied Sciences, Nanjing University, Nanjing 210023, China; 602024340071@smail.nju.edu.cn (H.Z.);

**Keywords:** photo-Fenton catalysis, Cu-FeOOH, oxygen vacancies, BiOCl nanoflowers, levofloxacin

## Abstract

Efficient photo-Fenton catalysis requires the simultaneous optimization of light harvesting, photogenerated-charge utilization, and metal-mediated H_2_O_2_ activation. Herein, amorphous Cu-FeOOH clusters were assembled on oxygen-vacancy-rich Fe-doped BiOCl nanoflowers (Fe-BOCov) to construct a Cu-FeOOH/Fe-BOCov composite for efficient photo-Fenton catalysis. Oxygen-vacancy engineering and Cu-FeOOH coupling extended visible-light absorption and improved interfacial charge separation, as supported by the enhanced photocurrent response and reduced electrochemical impedance. Under AM 1.5G simulated solar irradiation, the optimized catalyst removed more than 90% of levofloxacin (LEV) within 10 min and exhibited an apparent pseudo-first-order rate constant of 0.271 min^−1^. Radical-quenching and electron spin resonance measurements identify •OH and •O_2_^−^ as the principal reactive species, with photogenerated holes also contributing. A mechanism is proposed in which electrons generated in oxygen-vacancy BiOCl migrate to Cu-FeOOH sites, facilitate Fe(III)/Fe(II) and Cu(II)/Cu(I) cycling, and accelerate H_2_O_2_ activation. This work demonstrates a defect-and-interface strategy for coupling solar-energy utilization with bimetallic Fenton chemistry.

## 1. Introduction

Emerging contaminants, including pharmaceuticals, dyes, and nitroaromatic compounds [1,2,3], are increasingly prevalent in aquatic environments as a consequence of rapid industrialization and urbanization, posing serious threats to ecosystem integrity and human health [4,5]. Accordingly, diverse remediation technologies, including photocatalysis, adsorption, electrocatalysis, biodegradation, and Fenton-based oxidation, have been extensively investigated for wastewater treatment [5,6,7]. Among these approaches, photo-Fenton catalysis has attracted considerable attention because it integrates light harvesting with advanced oxidation, thereby enabling efficient degradation of organic pollutants under relatively mild conditions [8,9,10]. However, the practical implementation of photo-Fenton processes remains critically dependent on the development of catalytic materials with high structural stability, efficient charge utilization, and strong oxidant activation capability [11,12].

Layered bismuth oxyhalides (BiOX, X = Cl, Br, and I) have emerged as promising photocatalysts owing to their unique layered structures, tunable electronic properties, and favorable interfacial charge-transfer characteristics [13,14,15]. Among them, BiOCl has received considerable interest in heterogeneous photo-Fenton systems owing to its chemical stability and well-defined layered framework [16]. Nevertheless, pristine BiOCl suffers from limited visible-light utilization because of its relatively wide band gap, which restricts its photocatalytic efficiency [17]. Defect engineering, particularly the introduction of oxygen vacancies, has been demonstrated to be an effective strategy for overcoming this limitation [18,19]. Oxygen vacancies can induce defect states within the electronic structure [20,21], thereby narrowing the band gap and enhancing visible-light absorption [22]. More importantly, they can act as electron-trapping centers to facilitate charge separation and migration, thus improving photocatalytic performance [23].

In addition to defect modulation, heterointerface engineering provides another effective route to suppress the rapid recombination of photogenerated charge carriers [24]. When two semiconductors with different Fermi levels are coupled, interfacial charge redistribution can generate an internal electric field that drives directional separation of photoexcited electrons and holes. Amorphous FeOOH is regarded as a promising iron-based Fenton catalyst because of its small particle size and abundant redox-active Fe sites [25,26]. For example, Chang et al. [27] combined tubular carbon nitride with amorphous cobalt-doped FeOOH, significantly enhancing the degradation of sulfadiazine (SDZ), mainly due to accelerated interfacial electron transfer. However, FeOOH-based catalysts still suffer from several intrinsic drawbacks, including sluggish Fe(III)/Fe(II) cycling, strong dependence on acidic conditions, and the tendency of FeOOH clusters to aggregate, all of which limit their catalytic efficiency [28,29]. Recent studies have shown that Cu incorporation is an effective strategy for promoting the redox cycling of FeOOH-based catalysts [30]. Copper species can cooperate with Fe active centers to facilitate H_2_O_2_ activation and accelerate the Fe(III)/Fe(II) cycle, thereby improving overall catalytic activity. Xu et al. [31] synthesized Cu-doped α-FeOOH nanoflowers for diclofenac degradation and demonstrated that the enhanced performance originated from the synergistic activation of H_2_O_2_ by Cu and Fe species, as well as the Cu-promoted Fe redox cycle. These findings suggest that integrating Cu-modified FeOOH with vacancy-engineered BiOCl may provide a viable route for simultaneously enhancing visible-light harvesting, interfacial charge separation, and oxidant activation.

Here, we report amorphous Cu-FeOOH cluster-decorated porous oxygen-vacancy-rich Fe-doped BiOCl nanoflowers (Fe-BOCov) as an efficient 0D/3D photo-Fenton catalyst for antibiotic degradation. BiOCl is modulated by oxygen vacancy/Fe doping to enhance light capture and charge utilization; porous three-dimensional nanoflower frameworks are used to disperse and improve the accessibility of active sites; and amorphous Cu-FeOOH interface clusters are used to accelerate Fe(III)/Fe(II) and Cu(II)/Cu(I) redox cycles and H_2_O_2_ activation. Combined experimental characterizations reveal the interfacial charge-transfer behavior and enhanced photo-Fenton activity of Cu and Fe sites. In addition, radical-quenching experiments and EPR measurements were employed to elucidate the degradation mechanism of levofloxacin (LEV).

## 2. Materials and Methods

### 2.1. Materials

Cu(NO_3_)_2_⋅3H_2_O (AR), Fe(NO_3_)_3_⋅9H_2_O (AR), Na_2_SO_4_ (AR), Bi(NO_3_)_3_⋅5H_2_O (AR), NaCl (AR), polyvinylpyrrolidone (PVP, AR), and ethylene glycol (AR) were purchased from Shanghai Macklin Biochemical Technology Co., Ltd. (Shanghai, China) All reagents were used without further purification.

### 2.2. Preparation

Fe-doped BiOCl nanoflowers were first synthesized via a solvothermal method. Typically, 2 mmol Bi(NO_3_)_3_⋅5H_2_O was dissolved in 60 mL ethylene glycol (EG) under continuous stirring, followed by the addition of Fe(NO_3_)_3_⋅9H_2_O. The amount of Fe precursor was adjusted to obtain Fe/Bi molar ratios of 1%, 3%, 5%, and 7%, and the corresponding samples were denoted by 1%Fe-BiOCl, 3%Fe-BiOCl, 5%Fe-BiOCl, and 7%Fe-BiOCl, respectively. Subsequently, 2 mmol NaCl was added, and the mixture was stirred for 2 h until complete dissolution. The resulting suspension was transferred into a 100 mL Teflon-lined autoclave and maintained at 160 °C for 12 h. After naturally cooling to room temperature, the precipitate was collected by centrifugation, washed alternately with deionized water and ethanol, and dried at 60 °C for 12 h. For pristine BiOCl synthesis, no Fe(NO_3_)_3_⋅9H_2_O was added.

To construct the oxygen-vacancy-rich Fe-doped BiOCl sample, PVP was introduced during the solvothermal synthesis process. Typically, 2 mmol Bi(NO_3_)_3_⋅5H_2_O, 0.05 g PVP, and an appropriate amount of Fe(NO_3_)_3_⋅9H_2_O were added into 60 mL ethylene glycol (EG) under continuous stirring. The Fe/Bi molar ratio was fixed at 5%. The subsequent solvothermal treatment and post-treatment procedures were identical to those described above. The obtained oxygen-vacancy-rich Fe-doped BiOCl sample was denoted by Fe-BOCov. For comparison, BiOCl-ov was prepared using the same PVP-assisted solvothermal process without Fe(NO_3_)_3_⋅9H_2_O addition.

Cu-FeOOH/Fe-BOCov composites were synthesized by introducing Cu and Fe precursors onto the Fe-BOCov substrate. Typically, 0.2 g of Fe-BOCov, 1.2 g of Fe(NO_3_)_3_·9H_2_O, and different amounts of Cu(NO_3_)_2_⋅3H_2_O (0.1, 0.3, 0.6, and 0.9 mmol) were sequentially added into 100 mL of deionized water. After each addition, the suspension was ultrasonicated for 10 min to ensure uniform dispersion. The mixture was then heated in a water bath at 60 °C for 5 h. After cooling to room temperature, the products were collected by centrifugation, washed alternately with deionized water and ethanol, and dried in a vacuum oven at 60 °C for 12 h. The obtained samples were denoted by 0.1Cu-FeOOH/Fe-BOCov, 0.3Cu-FeOOH/Fe-BOCov, 0.6Cu-FeOOH/Fe-BOCov, and 0.9Cu-FeOOH/Fe-BOCov, corresponding to Cu precursor dosages of 0.1, 0.3, 0.6, and 0.9 mmol, respectively.

## 3. Results and Discussion

### 3.1. Structural Characterizations

The synthesis route is summarized in Figure 1a. To determine the crystal information and phase structure, the XRD patterns of BiOCl, Cu-FeOOH, and Cu-FeOOH/Fe-BOCov catalysts in Figure 1b–e were analyzed. Pristine BiOCl exhibits the reflections of tetragonal BiOCl (JCPDS No. 06-0249). Fe-BOCov exhibits characteristic peaks similar to those of BiOCl, and no additional Fe-related diffraction peaks were detected, which can be attributed to the relatively low Fe doping content [17]. Compared with pure BiOCl, the diffraction peaks of the (0 0 1) and (1 1 0) crystal planes of the Fe-doped composite material are slightly shifted (Figure 1b,d), which is attributed to the smaller radius of Fe^3+^ (0.65 Å) than that of Bi^3+^ (1.03 Å). This result indicates that Fe^3+^ has been successfully doped in situ into the BiOCl lattice [32,33]. Furthermore, some new characteristic peaks were observed in the XRD patterns of Fe-BOCov, located at 27.15°, 37.95°, 39.61°, and 48.69°, corresponding to the (012), (104), (110), and (202) crystal planes of metallic Bi. Notably, these diffraction peaks are relatively weak and broad, which may be due to Bi precipitation and the formation of oxygen vacancies [34]. The introduction of oxygen vacancies alters the thickness of BiOCl nanosheets, making Bi^3+^ more readily reduced to Bi^0^ [17] under PVP and thermal EG conditions. The diffraction peaks of the Cu-FeOOH clusters are relatively weak and broad, indicating that the clusters are amorphous. In the Cu-FeOOH/Fe-BOCov composite material, the intensity and width of this peak do not change significantly compared to the original Fe-BOCov, indicating that the basic structure of Fe-BOCov is not affected by the amorphous Cu-FeOOH clusters.

The morphology and structure of the prepared catalyst were observed using scanning electron microscopy (SEM). BiOCl has a sheet-like structure with nanosheets of various sizes stacked together. In Figure 1f,g,j,k BiOCl is assembled into a microsphere morphology by many nanosheets. Due to the special physical and chemical properties of ethylene glycol, thin nanosheets are formed in the ethylene glycol medium, and then the bent thinner nanosheets are guided to self-assemble into a 3D layered flower-like microsphere structure and grow along the (110) direction. As shown in Figure 1l, Cu-FeOOH clusters were successfully loaded onto the Fe-BOCov surface. The Cu-FeOOH/Fe-BOCov exhibits a porous nanoflower-like structure, indicating that the morphology of Fe-BOCov remains intact and closely connected after the introduction of Cu-FeOOH clusters, which is beneficial for carrier migration. EDS analysis showed that Fe and Cu elements were uniformly dispersed, and most Cu-FeOOH existed in the form of isolated nanoclusters.

Figure 2a–d shows the N_2_ adsorption–desorption isotherms and pore size distribution of BiOCl, 5%Fe-BiOCl, Fe-BOCov, FeOOH/Fe-BOCov, and 0.6Cu-FeOOH/Fe-BOCov. All samples exhibit type IV isotherms and type H3 hysteresis loops, indicating the presence of a mesoporous structure. The BET specific surface areas of Fe-BOCov and 0.6Cu-FeOOH/Fe-BOCov are 49.0 and 81.7 m^2^ g^−1^, respectively (Table S1). Compared to the original catalyst, 0.6Cu-FeOOH/Fe-BOCov exhibits a larger specific surface area. This increase in specific surface area can be attributed to the combined effects of oxygen vacancies and interfacial engineering, which contribute to increasing adsorption and surface reactive sites during the reaction process. The textural characteristics are expected to contribute to the enhanced reaction kinetics, although the catalytic improvement cannot be attributed to surface area alone.

The Raman spectra are shown in Figure 2e,f. Two significant characteristic peaks at 138 and 197 cm^−1^ are observed, representing Bi-Cl bands [18]. Characteristic peaks of FeOOH are observed at 212 and 272 cm^−1^, representing the amorphous Cu-FeOOH cluster [35,36]. All Cu-FeOOH/Fe-BOCov composites show similar results to the original Fe-BOCov, indicating that the loading of amorphous Cu-FeOOH clusters did not change the phase structure of Fe-BOCov.

FTIR spectroscopy was used to further reveal the chemical structure of the Cu-FeOOH/Fe-BOCov composite material and provide complementary information on surface functional groups. In Figure 2g,h, for the amorphous Cu-FeOOH clusters, the broad absorption peak at 2979–3500 cm^−1^ is attributed to the stretching vibration of O-H in Cu-FeOOH and the vibration of surface-adsorbed water [37]. The characteristic peak at 1407 cm^−1^ is related to the vibration of Cu-O. In addition, the absorption peak at 670–723 cm^−1^ is attributed to the out-of-plane bending vibration of Fe-O-H [38,39].

The surface chemical state of 0.6Cu-FeOOH/Fe-BOCov was further investigated using X-ray photoelectron spectroscopy (XPS). Full-scan spectra showed the coexistence of Cu, Fe, Bi, Cl, and O elements in the composite material (Figure 3a). The O 1s spectra exhibited three peaks at 529.58, 530.98, 531.88 eV, corresponding to lattice oxygen, OVs, and surface-adsorbed oxygen species (Figure 3b). In Figure 3c, the Fe 2p spectrum shows characteristic peaks at 710.2 and 713.71 eV, indicating the simultaneous presence of Fe^3+^ and Fe^2+^. The coexistence of different iron valence states is beneficial for electron transfer at the catalyst interface, which can promote the redox cycle activated by H_2_O_2_. In Figure 3d, the peak at 197.09 eV corresponds to Cl 2p_3/2_, while the peak at 199.53 eV belongs to Cl 2p_1/2_, indicating that chlorine in the catalyst exists in the form of Cl^−^.

The optical properties of the prepared samples were analyzed using UV-vis diffuse reflectance spectra (DRS). In Figure 3e, the introduction of oxygen vacancies and doping broadened the light absorption of BiOCl (from 362 nm to 518 nm to 550 nm). After successful loading of Cu-FeOOH clusters, the visible light absorption range of 0.6Cu-FeOOH/Fe-BOCov was extended to 608 nm. The absorption shoulder at 450–600 nm can be attributed to the interfacial charge transfer between BiOCl and Cu-FeOOH clusters. The band gaps of 0.6Cu-FeOOH and Fe-BOCov, calculated using the Kubelka–Munk method, are 1.90 eV and 2.25 eV (Figure 3f,g), respectively. Based on the XPS-VB spectra, Fe-BOCov is estimated to be 2.06 eV (Figure 3h). The shifts in the band positions after coupling suggest strong interfacial electronic interaction between BiOCl and Cu-FeOOH, indicating the formation of an intimate heterointerface rather than a simple physical mixture [40,41].

### 3.2. Photocatalytic Degradation Performance

Under AM 1.5G with H_2_O_2_ as the oxidant, the catalytic activity of the prepared samples for LEV degradation was evaluated. Before irradiation, the catalyst suspension was stirred in the dark for 40 min to establish adsorption–desorption equilibrium, followed by AM 1.5G irradiation. After the dark adsorption period, H_2_O_2_ was added to the reaction system to achieve a final concentration of 3 mM, and AM 1.5G irradiation was immediately initiated, which was defined as the starting point (t = 0 min) of the photo-Fenton reaction. As shown in Figure 4a,c, the Cu-FeOOH/Fe-BOCov composite material exhibited better LEV degradation performance than BiOCl and Cu-FeOOH. Among them, the 0.6Cu-FeOOH/Fe-BOCov composite material showed the highest catalytic activity, with a removal efficiency of over 90% after 10 min. The 0.6Cu-FeOOH/Fe-BOCov composite material achieved a LEV removal efficiency of 93.6% within 10 min, substantially higher than those of FeOOH (20.1%) and BiOCl (31.1%) under identical conditions. In Figure 4b, the 0.6Cu-FeOOH/Fe-BOCov composite material had the largest rate constant (0.271 min^−1^). Figure 4d shows that the LEV degradation rate increased as the nominal Fe/Bi molar ratio increased from 1% to 5%; however, the degradation efficiency decreased when the Fe/Bi molar ratio further increased to 7%. The 5% Fe-BiOCl sample achieved a 62.2% LEV removal efficiency within 10 min, while the removal efficiency of pure BiOCl was only 31.1%. For the Cu-FeOOH series (Figure 4e), the degradation efficiency gradually increased from 0.1Cu-FeOOH to 0.6Cu-FeOOH, while the degradation efficiency of 0.9Cu-FeOOH decreased. The 0.6Cu-FeOOH sample exhibited the highest activity in this series, achieving a 75.7% LEV removal efficiency within 10 min, while the removal efficiency of FeOOH was only 20.1%. These results highlight the importance of controlling the Fe incorporation and Cu addition amounts for improving catalytic performance. As summarized in Table S2, the optimized 0.6Cu-FeOOH/Fe-BOCov demonstrates competitive LEV degradation performance compared with recently reported heterogeneous photo-Fenton catalysts. Such enhanced activity originates from the rational integration of oxygen-deficient Fe-doped BiOCl and amorphous Cu-FeOOH clusters, rather than from a single modification. Compared with conventional Fe-based BiOCl systems, the introduced Cu-FeOOH clusters provide additional interfacial Cu/Fe redox sites, which facilitate the electron-transfer process involved in Fenton cycling and H_2_O_2_ activation. Meanwhile, unlike Cu-modified FeOOH catalysts that mainly depend on metal redox reactions, the BiOCl-based framework in this work contributes to light-driven charge generation and utilization. In addition, the three-dimensional nanoflower structure improves the accessibility of catalytic interfaces and promotes the interaction between LEV molecules, H_2_O_2_, and active sites. Therefore, the superior photo-Fenton performance of 0.6Cu-FeOOH/Fe-BOCov can be attributed to the synergistic coupling of photocatalytic and Fenton processes enabled by defect regulation, interfacial Cu/Fe chemistry, and hierarchical structural advantages.

The above results were further confirmed by transient photocurrent density under AM 1.5G irradiation and electrochemical impedance spectroscopy (EIS) (Figure 4f,g). Among them, 0.6Cu-FeOOH/Fe-BOCov exhibited the highest photocurrent response intensity and the smallest impedance radius. The stronger photocurrent response indicates more efficient separation and utilization of photogenerated carriers, whereas the smaller EIS semicircle suggests reduced interfacial charge-transfer resistance [42,43]. Therefore, 0.6Cu-FeOOH/Fe-BOCov achieved optimal photogenerated charge separation. When considered together with the broadened visible-light absorption and the mixed Fe valence states identified by XPS, these results are consistent with improved charge utilization and suggest that the transfer of photogenerated electrons to metal redox sites is facilitated. This proposed electron-transfer pathway may promote the regeneration of Fe(II) and Cu(I), thereby accelerating H_2_O_2_ activation.

In Figure 4h, the degradation efficiency decreased slightly after five cycles of centrifugation, washing, drying, and recycling, indicating that the Fe-BOCov, Cu-FeOOH, and 0.6Cu-FeOOH/Fe-BOCov composites all exhibit excellent recycling stability. The chemical structure changes of 0.6Cu-FeOOH/Fe-BOCov after five reaction cycles were determined by XRD. In Figure 4i, the crystal structure and phase composition of 0.6Cu-FeOOH/Fe-BOCov were not destroyed after 5 cycles, indicating that the observed pollutant removal mainly originates from catalytic oxidation rather than adsorption alone.

### 3.3. Catalytic Activity and Mechanisms

As shown in Figure 5a, by introducing disodium ethylenediaminetetraacetate (EDTA-2Na), isopropanol (IPA), and benzoquinone (BQ) as scavengers of holes (h^+^), •OH and •O_2_^−^, respectively, the major active species for LEV degradation in the photo-Fenton system were identified [44]. It can be seen that the presence of IPA and BQ significantly reduced the removal efficiency, indicating that •OH and •O_2_^−^ play crucial roles in the photo-Fenton degradation of LEV. The degradation performance decreased after the addition of EDTA-2Na, indicating that photogenerated holes are the active species, but their influence is less than that of •OH and •O_2_^−^. Therefore, it can be determined that the excellent photo-Fenton degradation performance is the result of the combined action of hydroxyl radicals, holes, and superoxide radicals. To further identify the reactive species involved in the photo-Fenton degradation process, EPR measurements were performed using DMPO as a spin-trapping agent. Under AM 1.5G simulated solar irradiation in the presence of H_2_O_2_, characteristic signals corresponding to DMPO-•O_2_^−^/DMPO-OOH and DMPO-•OH adducts were detected, indicating the generation of reactive oxygen species (ROS) during the catalytic reaction. As shown in Figure 5b, an apparent six-line EPR pattern was observed for the DMPO-•O_2_^−^/DMPO-OOH adduct under irradiation. The corresponding g factor was calculated to be approximately 2.0059. The apparent hyperfine coupling constants estimated from the experimental spectrum were A_N_ = 13.4 G and A_H_ = 8.8 G, which are comparable to the characteristic parameters reported for DMPO-OOH adducts. Although the smaller additional hyperfine splitting associated with DMPO-OOH was not fully resolved under the present experimental conditions, the observed splitting pattern, together with the characteristic EPR parameters, support the assignment of the signal to DMPO-•O_2_^−^/DMPO-OOH. Meanwhile, as shown in Figure 5c, the DMPO-•OH adduct exhibited the characteristic four-line splitting pattern with an intensity ratio of 1:2:2:1. The calculated g factor was approximately 2.0056, and the hyperfine coupling constants were determined to be A_N_ = A_H_ = 14.9 G. These values are consistent with the characteristic parameters reported for DMPO-•OH, further confirming the generation of hydroxyl radicals during the photo-Fenton reaction. The enhancement of the TEMPO signal under illumination suggests that h^+^ is involved in the photo-Fenton degradation process. Oxygen vacancies in Fe-doped BiOCl may facilitate the separation and transfer of photogenerated charge carriers. The photogenerated electrons may promote the reduction of Fe^3+^ and Cu^2+^ at the Cu-FeOOH sites, thereby regenerating Fe^2+^ and Cu^+^. These regenerated metal species can subsequently react with H_2_O_2_ through Fenton-like pathways to generate •OH and contribute to LEV degradation. Although the heterogeneous photo-Fenton mechanism was mainly investigated in this work, the influence of metal leaching on catalytic performance should be further evaluated to better understand the contribution of homogeneous reactions and the practical applicability of the catalysts.

Based on the experimental results, the possible degradation mechanism of LEV in the 0.6Cu-FeOOH/Fe-BOCov photo-Fenton system is proposed, as shown in Figure 5f. First, Fe-BOCov generates electrons and holes under AM 1.5G. The oxygen-vacancy-rich BiOCl substrate is proposed to facilitate interfacial photogenerated electron migration toward Cu-FeOOH active clusters, where the transferred electrons are expected to contribute to Fe^3+^/Fe^2+^ and Cu^2+^/Cu^+^ redox cycling [45]. At the same time, the presence of Fe^3+^/Fe^2+^ and Cu^2+^/Cu^+^ pairs can promote the generation of large amounts of hydroxyl radicals (•OH) from H_2_O_2_, thereby promoting the degradation of LEV. The existence of Cu^2+^/Cu^+^ not only serves as a reaction site to generate •OH, but also effectively promotes the Fe^3+^/Fe^2+^ cycle and maintains sufficient Fe^2+^ content in the system [46], thereby promoting the rapid generation of highly reactive •OH to achieve the degradation of LEV. During the degradation process, photogenerated h^+^ from Fe-BOCov and •O_2_^−^ generated from the reaction of photoelectrons with O_2_ also participate in the degradation of LEV to some extent. The heterogeneous structure of 0.6Cu-FeOOH/Fe-BOCov, through rational design, allows for synergistic effects between photocatalysis and the Fenton reaction, thereby improving catalytic efficiency and achieving highly efficient degradation of organic molecules. Furthermore, compared with the degradation efficiency of different catalysts for LEV in recent years, 0.6Cu-FeOOH/Fe-BOCov exhibits enhanced degradation performance (Figure 5e).

## 4. Conclusions

An amorphous Cu-FeOOH/Fe-BOCov composite was developed by depositing Cu–FeOOH clusters on porous, oxygen-deficient Fe-doped BiOCl nanoflowers. The nanoflower framework provides a high-surface-area and optically active scaffold, while the Cu-FeOOH clusters introduce dispersed Fe and Cu redox sites. The combined effects of defect engineering and interfacial coupling improve solar-light absorption, photogenerated-charge separation, and H_2_O_2_ activation. Under AM 1.5G simulated solar irradiation, the optimized composite achieved over 90% LEV removal within 10 min and exhibited an apparent pseudo-first-order rate constant of 0.271 min^−1^. Quenching and EPR measurements indicate that •OH and •O_2_^−^ are the dominant reactive species, with h^+^ also contributing to the degradation process. The enhanced activity can be attributed to the synergistic contributions of the individual components rather than solely to composite formation. Structural, spectroscopic, and electrochemical results collectively indicate that oxygen-deficient Fe-doped BiOCl improves solar-light harvesting and carrier utilization, while the amorphous Cu-FeOOH interface provides accessible bimetallic redox sites and promotes Fe(III)/Fe(II) and Cu(II)/Cu(I) cycling for H_2_O_2_ activation. The resulting coupling between photogenerated-carrier delivery and bimetallic Fenton chemistry accounts for the rapid LEV oxidation. Further research is needed under realistic water matrices to assess the practical applicability of this catalyst. Thus, this study demonstrates a defect-regulated and bimetallic interfacial strategy for enhancing photo-Fenton degradation of LEV.

## Figures and Tables

**Figure 1:**
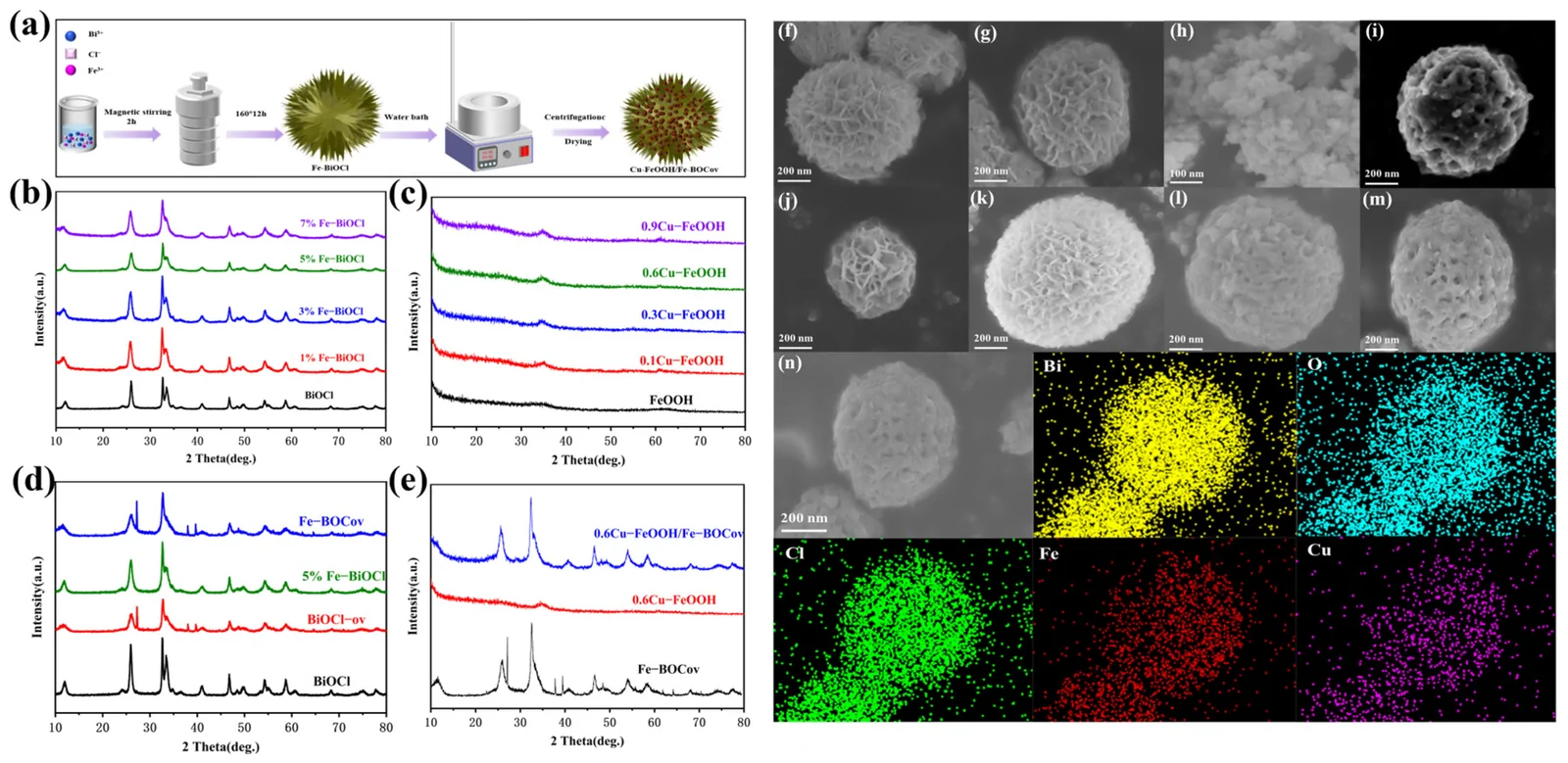
Schematic illustration and structural characterization of the prepared catalysts. (**a**) Schematic illustration of the synthesis process of 0.6Cu-FeOOH/Fe-BOCov. (**b**) XRD patterns of BiOCl and *x*%Fe-BiOCl (*x* = 1, 3, 5, and 7); (**c**) FeOOH and xCu-FeOOH (x = 0.1, 0.3, 0.6, and 0.9 mmol); (**d**) XRD patterns of BiOCl, BiOCl-ov, 5%Fe-BiOCl, and Fe-BOCov; (**e**) XRD patterns of Fe-BOCov, 0.6Cu-FeOOH, and 0.6Cu-FeOOH/Fe-BOCov. (**f**,**g**,**j**,**k**) SEM images of BiOCl; (**h**) SEM images of 0.6Cu-FeOOH and (**i**,**l**,**m**) SEM images of 0.6Cu-FeOOH/Fe-BOCov. (**n**) EDS elemental mappings of 0.6Cu-FeOOH/Fe-BOCov.

**Figure 2:**
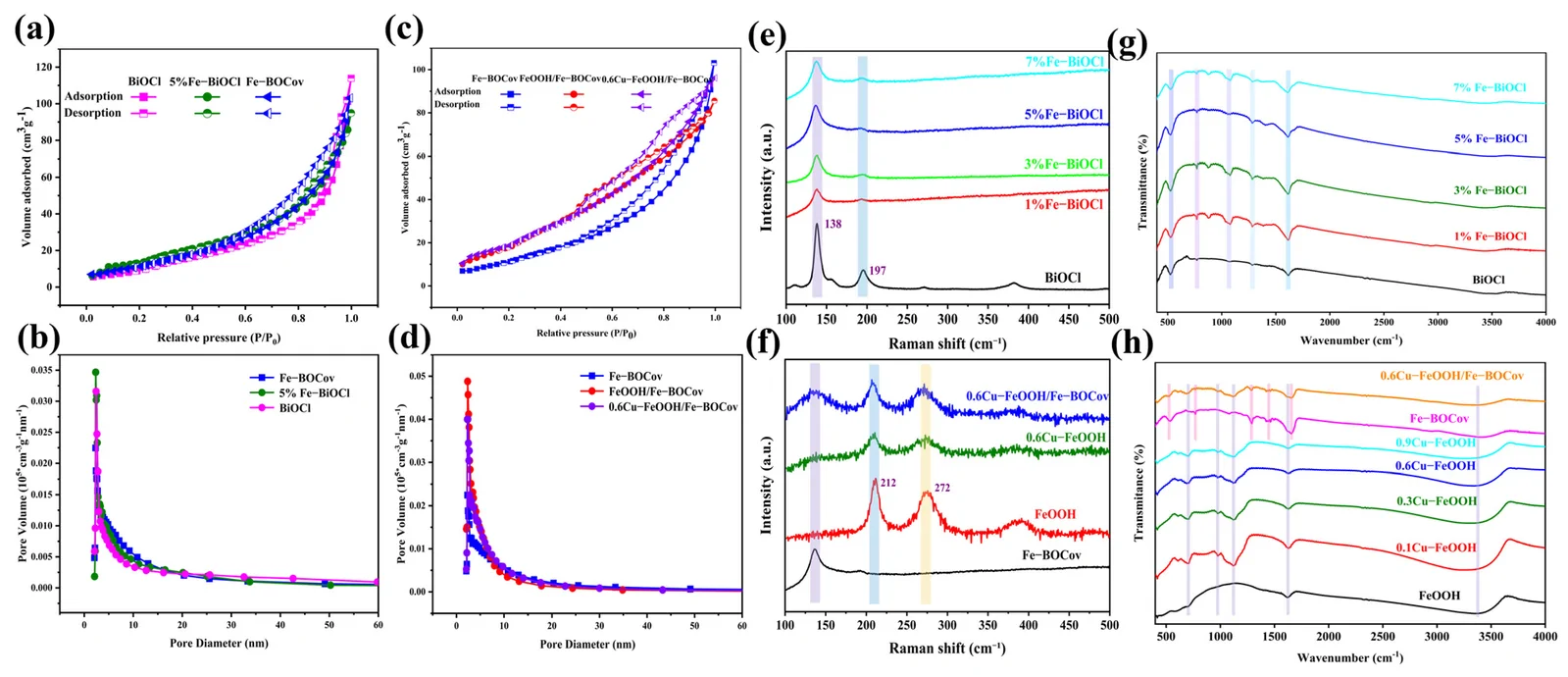
Textural and chemical structure characterization of the prepared catalysts. (**a**–**d**) N_2_ adsorption–desorption isotherms and corresponding pore size distributions of BiOCl, 5%Fe-BiOCl, Fe-BOCov, FeOOH/Fe-BOCov, and 0.6Cu-FeOOH/Fe-BOCov. (**e**,**f**) Raman spectra of the different samples. (**g**,**h**) FTIR spectra of the different samples.

**Figure 3:**
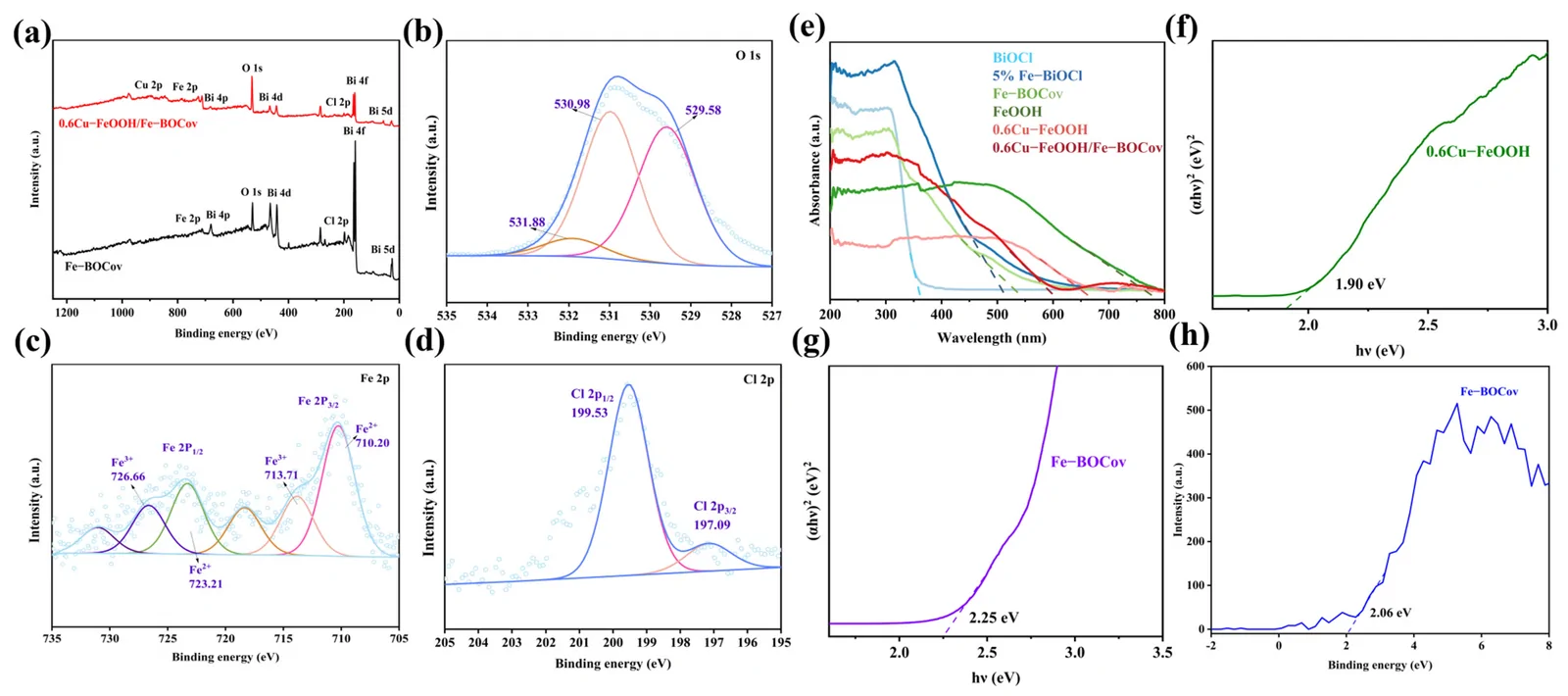
Surface chemical states and optical properties of the prepared catalysts. (**a**) XPS survey spectra. (**b**) O 1s XPS spectra. (**c**) Fe 2p XPS spectra. (**d**) Cl 2p XPS spectra. (**e**) UV–vis DRS. (**f**,**g**) Tauc plots of (αhν)^2^ versus hν for the determination of band gap energies. (**h**) XPS valence-band spectra of Fe-BOCov.

**Figure 4:**
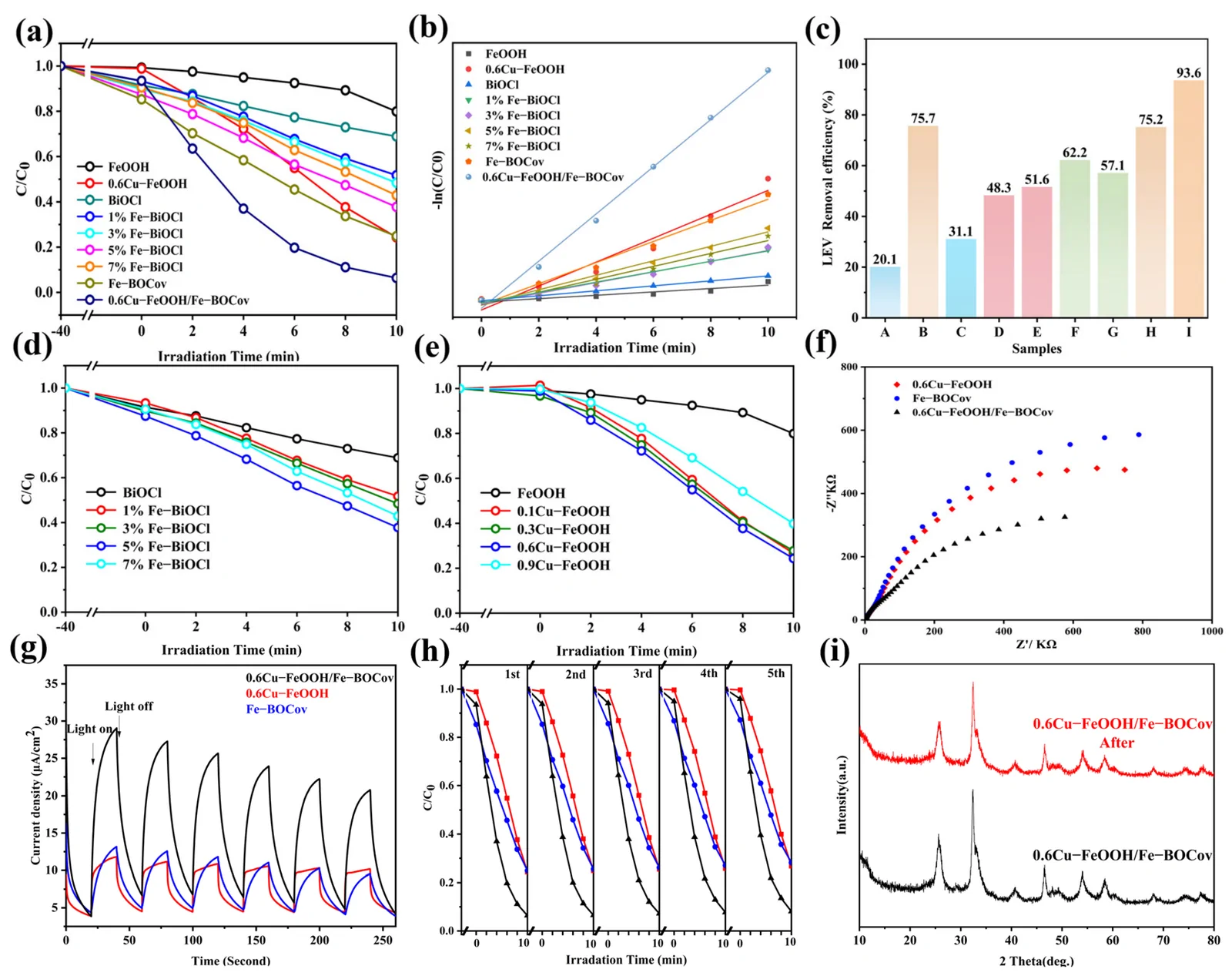
Photocatalytic degradation performance and electrochemical characterization of Fe-BOCov, Cu-FeOOH, and Cu-FeOOH/Fe-BOCov. (**a**) LEV degradation kinetics over different catalysts under simulated solar irradiation. (**b**) Pseudo-first-order kinetic plots for LEV degradation. (**c**) Comparison of LEV removal efficiencies among different catalysts (A: FeOOH, B: 0.6Cu-FeOOH, C: BiOCl, D: 1%Fe-BiOCl, E: 3%Fe-BiOCl, F: 5%Fe-BiOCl, G: 7%Fe-BiOCl, H: Fe-BOCov, I: 0.6Cu-FeOOH/Fe-BOCov). (**d**) LEV degradation profiles over BiOCl and Fe-doped BiOCl with different nominal Fe/Bi molar ratios. (**e**) LEV degradation profiles over FeOOH and Cu-FeOOH samples prepared with different Cu precursor amounts. (**f**) EIS Nyquist plots and (**g**) transient photocurrent responses under simulated solar irradiation. (**h**) Recycling stability of the catalysts after five consecutive cycles. (Black: 0.6Cu-FeOOH/Fe-BOCov, Red: 0.6Cu-FeOOH, Blue: Fe-BOCov) (**i**) XRD patterns of 0.6Cu-FeOOH/Fe-BOCov before and after cycling tests. All degradation experiments were performed with a catalyst dosage of 0.1 g L^−1^ and an initial LEV concentration of 10 mg L^−1^.

**Figure 5:**
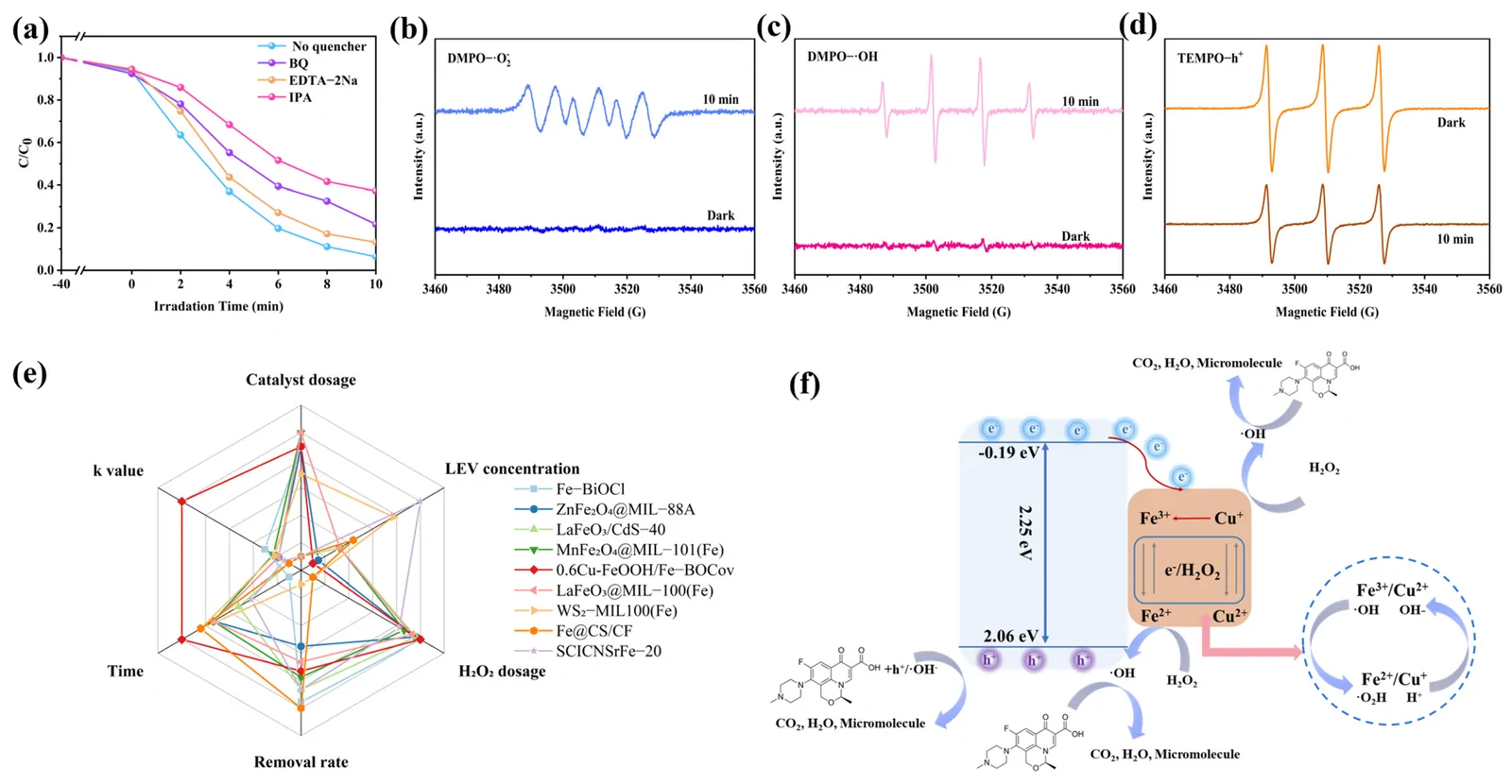
Investigation of reactive species and proposed photo-Fenton mechanism. (**a**) Effects of different scavengers on LEV degradation over 0.6Cu-FeOOH/Fe-BOCov. (**b**,**c**) EPR spectra of DMPO in the 0.6Cu-FeOOH/Fe-BOCov suspension under dark and after 10 min of irradiation. (**d**) EPR spectra of TEMPO in the 0.6Cu-FeOOH/Fe-BOCov suspension under dark conditions and after 10 min of irradiation. (**e**) Comparison of LEV degradation performance among different catalysts; detailed performance metrics are provided in Table S2 of Section S2 of the Supplementary Materials (the radar chart was generated based on min–max normalization, and all parameters were converted into dimensionless scores ranging from 0 to 10). (**f**) Schematic illustration of the proposed photo-Fenton degradation mechanism over 0.6Cu-FeOOH/Fe-BOCov.

## Data Availability

The original contributions presented in this study are included in the article and Supplementary Materials. Further inquiries can be directed to the corresponding authors.

## Supplementary Materials

The following supporting information can be downloaded at https://www.mdpi.com/article/10.3390/nano16191207/s1, Table S1: Textural properties of BiOCl, 5%Fe-BiOCl, Fe-BOCov, FeOOH/Fe-BOCov, and 0.6Cu-FeOOH/Fe-BOCov. Table S2: Comparison of reported photo-Fenton catalysts for LEV degradation. Table S3. Sample designations and their definitions. References [17,47,48,49,50,51,52,53,54] are cited in the Supplementary Materials.
