# Supplementary material for: Amorphous Cu-FeOOH Clusters on Oxygen-Deficient Fe-Doped BiOCl Nanoflowers for Efficient Photo-Fenton Degradation of Levofloxacin
Source: Nanomaterials (Basel). 2026 Sep 24;16(19):1207. doi: 10.3390/nano16191207 (PMC13667265; doi:10.3390/nano16191207)
Supplement: Supplementary file 1 [file nanomaterials-16-01207-s001.zip › nanomaterials-4550089-supplementary.pdf]

Supplementary Materials

Amorphous Cu-FeOOH Clusters on Oxygen-Deficient Fe-Doped BiOCl Nanoflowers for Efficient Photo-Fenton Degradation of Levofloxacin

Hailin Zhang [a,†], Jin Cao [a,†], Xin Liu [a], Di Wei [a], Shuangpeng Yan [a], Xiangmei Wang [a], Hao Liu [a], Han Shi [a], Xinkun Ren [a,*], Shanyuan Niu [a,*]

a College of Engineering and Applied Sciences, Jiangsu Key Laboratory of Functional Metamaterials, Nanjing University, Nanjing 210023, China

*Corresponding author: nius@nju.edu.cn (S.N.); xinkunren@nju.edu.cn (X.R.)

† These authors contributed equally to this work.

S1. Materials and Methods

S1.1. Characterization

The phase composition and crystal structure of the samples were analyzed by X-ray diffraction (XRD; D8 ADVANCE, Bruker AXS GmbH, Karlsruhe, Germany) using Cu Kα radiation (λ = 0.15406 nm) over a 2θ range of 10-80°, with an operating voltage of 40 kV and a current of 40 mA. The surface elemental composition and chemical states of the catalysts were analyzed by high-resolution X-ray photoelectron spectroscopy (XPS; K-Alpha Nexsa, Thermo Fisher Scientific, Waltham, MA, USA) using a monochromatic Al Kα X-ray source (hν = 1486.6 eV). The binding energies were calibrated against the C 1s peak at 284.8 eV. Brunauer–Emmett–Teller (BET) specific surface areas and pore-size distributions were measured using an automatic surface-area and porosity analyzer (JW-BK200, Beijing JWGB Instruments Co., Ltd., Beijing, China) from N_2 adsorption at 77 K. Before measurement, the samples were degassed at 100℃ for 8h under vacuum flow. Sample morphologies were observed by scanning electron microscopy (SEM, Carl Zeiss, Oberkochen, Germany) at an accelerating voltage of 5 kV. UV-vis diffuse-reflectance spectra (DRS) were recorded using a UV-3600 UV–vis spectrophotometer (Shimadzu Corporation, Kyoto, Japan) over 200-800 nm with $BaSO_4$ used as the reflectance reference. The optical band-gap energies were estimated from the corresponding Tauc plots. Fourier transform infrared (FTIR) spectra were recorded using an INVENIO spectrometer (Bruker) over the wavenumber range of 500-4000 cm^{-1} using the KBr pellet mode. Raman spectra were collected using an iHR550, HORIBA Raman spectrometer, Raman-shift range of 100-500 cm^{-1}. Photoelectrochemical measurements were carried out on a CHI-760E electrochemical workstation (Chenhua, Shanghai, China) using catalyst-coated FTO glass as the working electrode, a Pt wire as the counter electrode, and saturated Ag/AgCl as the reference electrode. Photocurrent responses were recorded under AM 1.5G irradiation from a 300 W xenon lamp (CEL-HXF300-S). Electron paramagnetic resonance (EPR) spectra were measured using a Bruker EMXplus-6/1. 5,5-Dimethyl-1-pyrroline N-oxide (DMPO) was used as the spin-trapping agent for the detection of •OH and $•O_2^-$ radicals.

S1.2. Photo-Fenton Degradation Experiments

An aqueous levofloxacin (LEV) solution was used as the model pollutant. In a typical experiment, 10 mg of catalyst was dispersed in 100 mL of LEV solution (10 mg L^{-1}). Before irradiation, the suspension was magnetically stirred in the dark for 40 min to establish adsorption–desorption equilibrium. After the dark adsorption period, H_2O_2 was added to the reaction suspension to achieve a final concentration of 3 mM. The AM 1.5G simulated solar irradiation was then immediately initiated, and this point was defined as t = 0 min.

During irradiation with a 300 W xenon lamp (CEL-HXF300-S), the reactor was cooled in an ice-water bath and continuously stirred to maintain a homogeneous catalyst suspension. The residual LEV concentration was monitored by UV–vis spectrophotometry using the absorption at 290 nm.

S2. Supplementary Tables

Table S1. Textural properties of BiOCl, 5%Fe-BiOCl, Fe-BOCov, FeOOH/Fe-BOCov, and 0.6Cu-FeOOH/Fe-BOCov.

Sample	S_BET ($m^2\ g^{-1}$)	V_pore ($cm^3\ g^{-1}$)	D_pore (nm)
BiOCl	42.6	0.196	15.233
5%Fe-BiOCl	55.8	0.163	9.825
Fe-BOCov	49.0	0.180	12.436
FeOOH/Fe-BOCov	77.0	0.160	6.343
0.6Cu-FeOOH/Fe-BOCov	81.7	0.172	7.603

Table S2. Comparison of reported photo-Fenton catalysts for LEV degradation.

Catalyst	Catalyst dosage	[LEV] (mg L^{-1})	[H_2O_2] (mM)	Removal efficiency (time)	k_app (min^{-1})	Ref.
Fe-BiOCl	10 mg	10	3	99% (180 min)	0.07100	[17]
$ZnFe_2O_4$@MIL-88A	10 mg	12	4.9	90% (60 min)	0.03828	[47]
$LaFeO_3$/CdS-40	50 mg	20	5	97% (100 min)	0.01260	[48]
$MnFe_2O_4$@MIL-101(Fe)	5 mg	20	–	95% (60 min)	0.05056	[49]
Ag/AgCl/Bi_2O_3/$BiFeO_3$	100 mg	18	–	72% (90 min)	–	[50]
$LaFeO_3$@MIL-100(Fe)	5 mg	20	6.625	92.5% (60 min)	0.03810	[51]
WS_2-MIL100(Fe)	20 mg	40	48.75	80% (40 min)	0.04756	[52]
Fe@CS/CF	50 mg	25	49	100% (40 min)	0.01310	[53]
SClCNSrFe-20	50 mg	50	8.8	97% (120 min)	0.02410	[54]
0.6Cu-FeOOH/Fe-BOCov	10 mg	10	3	94% (10 min)	0.27059	This work

Notes. The radar chart was constructed based on six criteria, including degradation efficiency, reaction kinetics, catalyst stability, H_2O_2 activation capability, mechanistic elucidation, and practical applicability. Quantitative parameters were evaluated according to the reported experimental results and converted into normalized scores using the min–max normalization method. Specifically, degradation efficiency was scored according to pollutant removal efficiency (<50%, 50–70%, 70–85%, 85–95%, and >95% corresponding to 2, 4, 6, 8, and 10 points, respectively), while reaction kinetics was evaluated based on the apparent pseudo-first-order rate constant (k < 0.01, 0.01–0.05, 0.05–0.10, 0.10–0.20, and >0.20 min^{-1} corresponding to 2, 4, 6, 8, and 10 points, respectively). Catalyst stability was evaluated according to the retained catalytic activity after recycling tests (<70%, 70–80%, 80–90%, and >90% retention after repeated cycles corresponding to increasing scores). H_2O_2 activation capability was assessed based on the reported evidence of H_2O_2-dependent degradation, reactive oxygen species identification, and Fe/Cu redox cycling. Mechanistic elucidation was evaluated according to the depth of mechanistic analysis, including radical scavenging experiments, EPR spectroscopy, XPS valence-state analysis, electrochemical measurements, and charge-transfer investigation. Practical applicability was evaluated considering catalyst recyclability, tolerance toward coexisting ions, real-water validation, metal-leaching analysis, and long-term stability. The scores of these semi-quantitative criteria were subsequently normalized for radar-chart visualization. Because the reaction conditions, including catalyst dosage, pollutant concentration, irradiation source, H_2O_2 concentration, and reaction time, vary among different studies, the radar chart is intended to provide a semi-quantitative comparison of catalytic characteristics rather than an absolute ranking of catalyst performance.

Table S3. Sample designations and their definitions.

Sample designation	Description and definition
BiOCl	Pristine BiOCl reference sample.
1%Fe-BiOCl	Fe-doped BiOCl with a nominal Fe/Bi feed molar ratio of 1%.
3%Fe-BiOCl	Fe-doped BiOCl with a nominal Fe/Bi feed molar ratio of 3%.
5%Fe-BiOCl	Fe-doped BiOCl with a nominal Fe/Bi feed molar ratio of 5%.
7%Fe-BiOCl	Fe-doped BiOCl with a nominal Fe/Bi feed molar ratio of 7%.
BiOCl-ov	Oxygen-vacancy-rich BiOCl prepared without an Fe precursor.
Fe-BOCov	Oxygen-vacancy-rich Fe-doped BiOCl with a nominal Fe/Bi feed molar ratio of 5%; the support used for composite synthesis.
FeOOH	FeOOH prepared without Cu addition.
0.1Cu-FeOOH	Unsupported Cu-modified FeOOH prepared with 0.1 mmol of Cu precursor.
0.3Cu-FeOOH	Unsupported Cu-modified FeOOH prepared with 0.3 mmol of Cu precursor.
0.6Cu-FeOOH	Unsupported Cu-modified FeOOH prepared with 0.6 mmol of Cu precursor.
0.9Cu-FeOOH	Unsupported Cu-modified FeOOH prepared with 0.9 mmol of Cu precursor.
FeOOH/Fe-BOCov	FeOOH deposited on Fe-BOCov without Cu addition.
0.1Cu-FeOOH/Fe-BOCov	Cu-FeOOH/Fe-BOCov composite prepared with 0.1 mmol of Cu precursor.
0.3Cu-FeOOH/Fe-BOCov	Cu-FeOOH/Fe-BOCov composite prepared with 0.3 mmol of Cu precursor.
0.6Cu-FeOOH/Fe-BOCov	Cu-FeOOH/Fe-BOCov composite prepared with 0.6 mmol of Cu precursor; the best-performing composite in this study.
0.9Cu-FeOOH/Fe-BOCov	Cu-FeOOH/Fe-BOCov composite prepared with 0.9 mmol of Cu precursor.

Notes. The percentages preceding Fe denote nominal Fe/Bi feed molar ratios calculated from the precursor amounts, rather than measured Fe contents in the products.

These prefixes are sample identifiers, not Cu weight or molar percentages. The Cu precursor is $Cu(NO_3)_2{\cdot}3H_2O$.
